# Psychological hardiness and social support as protective factors against burnout in high-performance athletes

**DOI:** 10.3389/fspor.2025.1726003

**Published:** 2025-12-18

**Authors:** Daniel Oleas, David Alarcón Rubio, María del Pilar Méndez-Sánchez, Manuel Trujillo, José Carlos Jaenes

**Affiliations:** 1 Department of Research, Universidad Ecotec, Samborondón, Ecuador; 2 Department of Social Anthropology, Basic Psychology and Public Health, Pablo de Olavide University, Seville, Spain; 3 Faculty of Higher Studies Zaragoza, National Autonomous University of Mexico, Mexico City, Mexico; 4 Grossman School of Medicine, New York University, New York, NY, United States

**Keywords:** athlete burnout, competition level, hardiness, high-performance athletes, social support, stress adaptation

## Abstract

Burnout Syndrome (BOS) is a psychological condition characterized by emotional exhaustion, depersonalization, and a reduced sense of accomplishment, frequently observed in high-performance athletes exposed to chronic stress and competitive pressure. This study examined the role of hardiness and social support as potential protective factors against burnout and its dimensions while accounting for demographic and sports-related variables such as age, sex, and level of competition. The sample comprised 388 high-performance athletes aged between 15 and 45 years (46% women; *M* = 27.31, SD = 8.51). Participants completed the validated Spanish versions of the Athlete Burnout Questionnaire (ABQ), Resilient Personality Scale for Marathoners (EPRM), and Social Support Scale (EAS). The data were analyzed using nonparametric tests (Kruskal–Wallis test) and multiple linear regression models. The results revealed that sex was not a significant predictor of burnout, whereas age and competition level were negatively associated with overall burnout scores. Among the psychological variables, hardiness, particularly commitment and control dimensions, emerged as the strongest protective factor, whereas social support was negatively associated with emotional exhaustion and total burnout. The significant interaction between social support and competition level indicated that younger athletes had a stronger protective effect than senior athletes. These results show that social support plays a different role in competitive stages, indicating early reinforcement of athletes' social networks can significantly lower their burnout risk. Practical implications for coaches and sports psychologists in developing integrated prevention and intervention programs that enhance athletes' hardiness and social connection throughout their careers.

## 1 Introduction

Following the 2020 Tokyo and Paris 2024 Olympic Games, an increasing number of athletes have expressed serious doubts about participating in the next Olympic cycle. World, Olympic, and European champions began considering retirement. This trend is not attributable to physical decline or age but rather to structural factors. These include the pressure of high-performance demands and the perceived lack of support from federations, clubs, or even coaches, which often contributes to the state of uncertainty (1); an emotional state compounded when sports do not provide long-term financial security (2). This lack of stability often leads athletes to devalue their sport, experience emotional detachment, and view the conclusion of major events as the start of a life stage for which they are unprepared (3). This problem is further exacerbated by the limited support provided by sports to help athletes facing uncertain futures alone (4). All these factors contribute to sports abandonment as a highly probable outcome, especially among athletes with high success expectations.

In this context, burnout syndrome has emerged as a key construct for understanding the progressive exhaustion experienced by high-performance athletes. Freudenberger (5) initially described it as a process of psychiatric and physical breakdown that primarily affects dedicated and committed workers in helping professions, who often neglect their own needs in favor of others. Currently, burnout syndrome is not limited to helping professions but extends to all types of workers and individuals across various contexts (6), including sports (7).

Contemporary research has established burnout as a multidimensional condition encompassing emotional and physiological responses to prolonged exposure to stressors, comprising three key components: 1) overwhelming exhaustion with feelings of weariness, loss of energy, depletion, and fatigue; 2) cynicism and detachment with depersonalization, negative attitudes, irritability, withdrawal from the sport, and a sense of ineffectiveness; and 3) lack of accomplishment and an inability to cope with or confront challenges (8).

From a pathophysiological and pathogenesis view of burnout syndrome, Weber and Jaekel-Reinhard (9) examined whether burnout represents “a disease of modern societies” and established a link with mental health by associating it with chronic unresolved high-level stress. Bianchi and Schonfeld (10) later highlighted a significant overlap between burnout and pathology. They concluded that burnout is better understood as a form of depression than as a distinct clinical entity.

This growing recognition of burnout has also extended to the field of sports. Burnout occurs when athletes perform at a high level under extreme stress and pressure. The accumulation of these stressors can eventually exceed coping capacity, resulting in burnout (11). This phenomenon occurs most often in elite performance but can occur to anyone (12). This phenomenon is prevalent in various sports, including elite football, where it affects between 3.4% and 3.8% of the athletes in Spain (13). The stress that causes burnout can come from many stressors in sports, including overtraining, internal pressure (perfectionism), external sources (coaches, family members, etc.), and high expectations (14). To decrease the risk of burnout, an athlete must practice healthy habits such as getting good sleep, eating balanced meals, and dedicating time to enjoyable activities (15).

Hardiness, first introduced by Kobasa (16), is a trait associated with performance under stressful conditions. It refers to a personal worldview that encompasses the ability to adaptively respond to challenging situations and forms the psychological foundation of resilience (17). In different studies, hardiness was found to predict health and positive behavioral outcomes during difficult times, such as the COVID-19 lockdown (18). During this period, many athletes reported difficulties in maintaining their psychological well-being (19–21). Higher levels of hardiness have been linked to greater sports autonomy and resilience (22). Hardiness is associated with lower burnout rates in workers and acts as a protective factor for both men and women in demanding environments (23). Similarly, hardiness training programs, such as those implemented in nursing, have demonstrated effectiveness in managing stress and reducing burnout in health care settings (24).

Hardiness comprises three key dimensions: 1) commitment, that is, actively engaging in activities and social interactions rather than feeling alienated; 2) control, the belief that one can influence life situations, shaped by learning and experience; and 3) challenge, viewing change as an opportunity for growth rather than a threat to security (25). In sports, marathon runners exhibit higher levels of hardiness than university students (26). Other research has identified significant differences in psychological hardiness between female student athletes in individual sports and those participating in team sports, with individual sports athletes demonstrating greater competitiveness and mental toughness (27). Furthermore, hardiness has been positively correlated with sporting achievement and psychological well-being and negatively associated with psychological distress (28), suggesting its role in predicting mental health and performance in sports.

Both burnout and hardiness are psychological constructs that are relevant across various professional contexts and extend beyond the athletic domain. These constructs are particularly prominent in high-stress occupations, such as nursing and medicine, where both men and women are exposed to intense emotional and physical demands (29, 30). Hardiness can function as a protective factor in such environments, and the prevalence of burnout underscores the need for targeted psychological interventions.

Social support refers to the positive effects of social interactions that help people cope with difficulties and mitigate the health impact of stressors. In sports, the support of parents, coaches, and teammates is essential in various situations, including competitions, injuries, and personal challenges. Numerous studies have emphasized the role of sleep in maintaining both athletic performance and psychological well-being (31–33). Social support includes financial, emotional, tactical, and strategic support, with coach support plays a particularly critical role in moderating the stress-burnout relationship (34). However, social support should be interpreted as a complementary resource that enhances, but does not replace, internal commitment and resilience capacities.

Bianco and Eklund (35) conducted a foundational research on social support in the context of sports-related injuries. According to the authors, it is a broad concept that includes different types of interventions and interactions, including shared activities, various forms of interpersonal communication, and emotional messaging. This multifaceted nature highlights its potential as a protective factor against burnout in high-performance sports.

The triad of burnout, hardiness, and social support is a recurring theme in contemporary research, with relevance across work, education, and sports. In athletic contexts, both burnout and psychological resilience—often operationalized through hard work—are observable at all levels of competition and roles in sports. Hardiness is generally regarded as a protective trait that enhances adaptability, whereas strong social support networks contribute to emotional stability and sustained performance. On the contrary, burnout continues to be a prevalent issue that compromises athletes' well-being and professional longevity. Although these constructs have been widely studied, particularly in occupational contexts [see, e.g., (36)], their joint impact within high-performance sports settings remains underexplored.

The general aim of this study was to explore the relationships between athlete burnout components and key psychological and sport-related variables, including psychological hardiness, perceived social support, age, and competitive category in high-performance athletes. Specifically, this research sought to explore the influence of age and competitive category on burnout components; analyze the associations between psychological hardiness (commitment, control, and challenge) and burnout indicators; examine the relationship between perceived social support and burnout; and evaluate whether competitive category moderates the association between perceived social support and athlete burnout components.

This study contributes to the development of practical intervention protocols aimed at preventing and managing athlete burnout and reducing premature sports abandonment. Previous studies have shown improvements in key areas, including emotional exhaustion, depersonalization, personal achievement, and overall mental well-being (15, 37). These results are promising and could be tested among athletes, coaches, and students to assess their potential for strengthening hardiness and reducing burnout through social support.

## 2 Methodology

### 2.1 Subjects

The study included 388 high-performance athletes, with a mean age of 27.31 years (SD = 8.51), ranging from 15 to 45 years. The sample comprised 180 (46%) women and 208 (54%) men. The athletes were divided into two categories according to the total sample: 37% (*n* = 142) were classified as youth athletes, while 63% (*n* = 226) belonged to the classification at the competition level of the senior group, following the Spanish Royal Track and Field Federation: youth athletes (under 23 years old) and senior athletes (23 years and older). A cross-tabulation of age groups and sex was performed to further describe the sample.Among the youth athletes, 76 (20% of the total sample) and 66 (17%) were men.In the senior group, 132 were men (34%) and 114 were women (29). Weekly training hours varied among participants: 18% trained up to 5 h per week, 54% trained between 6 and 10 h, 21% trained between 11 and 15 h, and 7% trained for more than 15 h per week. Finally, 191 athletes reported their educational level and responses were grouped into three categories: (1) primary and secondary education (*n* = 55; 15%), (2) technical or vocational education (*n* = 33; 9%) and (3) higher education (university or postgraduate) (*n* = 103; 28%).

### 2.2 Instruments

This study used a validated Spanish version (38) of The Athlete Burnout Questionnaire (ABQ) (39) to assess burnout. The ABQ comprises 15 items that measure three burnout dimensions in sports: reduced sense of accomplishment (feelings of fatigue and depletion derived from sport demands), emotional exhaustion (a negative and detached attitude toward sport participation), and reduced personal achievement (perceptions of declining performance and achievement). Responses were recorded on a 5-point Likert scale ranging from (1) hardly ever to (5) almost always, with higher scores reflecting greater burnout severity. The ABQ demonstrated robust internal consistency, as evidenced by Cronbach's *α* coefficients ranging from .71 to .87.

The Spanish version of the Scale of Resilient Personality (Hardiness) in Marathoners (EPRM) (25) was used to measure the participants' hardness. The EPRM contains 30 items, with 10 questions assessing each of the three dimensions: commitment (the tendency to remain engaged and find meaning in sport and daily activities rather than feeling apathetic or alienated), challenge (the perception of change and competitive demands as opportunities for personal growth rather than as threats), and control (the belief that one can influence outcomes and effectively manage sport-related challenges instead of feeling powerless). Items are rated on a 4-point Likert scale ranging from 1 (totally agree) to 4 (totally disagree), with higher scores indicating greater levels of psychological hardiness. The EPMR demonstrated acceptable internal consistency, as evidenced by a Cronbach's *α* coefficient of .79.

The Social Support Scale (EAS), originally developed by Cresswell and Eklund (40) and Cresswell (41) and validated in Spanish (42), was used in this study. The EAS comprises five questions that assess perceived support in response to positive and negative sporting outcomes. The questions explore whether the athlete is receiving support in different situations: when the athlete receives positive or negative results, when they feel frustrated, or after undergoing very demanding moments. Responses are rated on a 5-point Likert scale ranging from 1 (almost never) to 5 (almost always), with higher scores indicating greater perceived social support. Social support has historically been recognized as a key moderator of life stress (71). The internal consistency of the scale was high with *α* .88

Demographic data, including age, sex, competition level, and weekly training hours, were also collected.

### 2.3 Procedure

This study employed a descriptive quantitative methodology using random non-purposive sampling and the snowball effect. An online *ad hoc* questionnaire was distributed among competitive Spanish athletes, incorporating demographic questions, research objectives, and ethical considerations. Participants received detailed instructions for completing the questionnaire, along with informed consent for adults and parental permission for athletes aged <18 years of age. The questionnaire was distributed through mainstream digital platforms, including the Internet and WhatsApp, as validated methods for data collection (43, 44).

### 2.4 Data analysis

All statistical analyzes were conducted using Jamovi version 2.26 (45). Before the main analyses, the Kolmogorov–Smirnov test was used to evaluate the assumption of normality, which indicated that the distributions of the assumptions of variables violated the normality (*p* < .001). Consequently, nonparametric tests were employed where appropriate for the data.

Kruskal–Wallis H test was used to examine group differences between sex and competition level (youth vs. Senior athletes). This test is a robust nonparametric alternative to one-way analysis of variance (ANOVA) and is suitable for comparing two or more independent groups when the data are not normally distributed or when the variances are unequal (46). Although originally designed for more than two groups, the Kruskal–Wallis test can be validly applied to two-group comparisons, offering consistency in the analytical strategy across variables and categories. The test compares the distribution of ranks across groups and is particularly useful when dealing with ordinal or Likert-type scales.

The relationships between age, perceived social support, burnout components, and hardiness traits were examined using Spearman's rank order correlation, which is appropriate for ordinal and continuous non-normally distributed variables and does not require assumptions of normality. To test the predictive capacity of hardiness, social support, age, sex, and level of competition on burnout outcomes, four multiple linear regression models were performed using the enter method. Each model predicted one outcome: reduced sense of accomplishment, emotional exhaustion, evaluation, and total burnout. Independent variables included the three hardiness (control, commitment, and challenge), perceived social support, chronological age, sex (reference: male) and competition level (reference: youth athletes). Furthermore, an interaction term between competition level and social support was used was used to assess whether the impact of social support on burnout differed between age-based competition levels.

## 3 Results

### 3.1 Descriptive statistics

Table 1 summarizes the descriptive statistics and the differences between the sex and competition level for each variable analyzed. The descriptive analyzes included the mean and standard deviation values. Given the nonnormal distribution of the data, the Kruskal–Wallis test was used to compare differences between sex and between competitive categories (youth vs. senior). This non-parametric test is a robust statistical approach when normal assumptions are violated.

**Table 1:** Descriptive statistics and sex differences.

Variables	Young				Senior				*X*^2^ (Category)	*X*^2^ (Sex)
	Male (*n* = 76)		Female (*n* = 66)		Male (*n* = 132)		Female (*n* = 114)			
	Media	DE	Media	DE	Media	DE	Media	DE		
Control (EPRM)	23.97	2.89	23.48	2.84	23.90	2.80	24.18	2.47	1.02	0.11
Commitment (EPRM)	23.03	2.66	23.24	2.47	23.36	2.83	23.90	2.23	6.10*	1.31
Challenge (EPRM)	20.59	2.94	20.71	3.23	20.40	3.13	20.68	3.01	0.17	0.70
Social Support	19.68	4.47	21.59	3.81	20.27	4.44	20.46	4.30	0.17	3.22
Reduced sense of accomplishment	13.04	2.39	13.05	2.16	12.03	2.44	11.64	2.12	23.07***	0.83
Emotional exhaustion	10.32	3.20	10.61	3.49	9.25	2.72	9.54	2.83	9.13**	0.63
Devaluation	13.07	2.75	12.53	2.83	12.37	2.92	11.93	2.71	4.56*	3.68
Burnout	36.42	6.72	36.18	6.93	33.65	6.14	33.11	6.06	18.50***	0.67

*X*^2^, Kruskal–Wallis.

* *p* < .05.

** *p* < .01.

*** *p* < .001.

### 3.2 Relationship between social support, burnout, hardy personality, and Age

Spearman rank-order correlations were used to assess the relationships among age, social support, burnout dimensions, and hardiness traits. Table 2 shows significant correlations were observed between most variables. Age was negatively associated with burnout components. Social support was negatively correlated with overall burnout and emotional exhaustion. All three hardiness traits, control, commitment, and challenge, were significantly correlated with lower levels of burnout.

**Table 2:** Spearman correlations.

Variable	1.	2.	3.	4.	5.	6.	7.	8.
1. Age	—							
2. Control (EPRM)	0.07	—						
3. Commitment (EPRM)	0.09	0.33**	—					
4. Challenge (EPRM)	−0.06	0.33**	0.32**	—				
5. Social support	−0.05	0.20**	0.17*	0.18**	—			
6. Reduced sense of accomplishment	−0.25**	−0.32**	−0.28**	−0.22**	−0.16*	—		
7. Emotional exhaustion	−0.16*	−0.38**	−0.33**	−0.29**	−0.24**	0.53**	—	
8. Devaluation	−0.08	−0.21**	−0.26**	−0.13*	−0.09	0.18**	0.51**	—
9. Burnout	−0.22**	−0.38**	−0.36**	−0.26**	−0.20**	0.69**	0.88**	0.73**

* *p* < .01.

**p < .001.

### 3.3 Multiple linear regression models

Four multiple linear regression models using the enter method were used to identify the predictors of reduced sense of achievement, emotional exhaustion, devaluation, and Burnout. The models included the following independent variables: three dimensions of hardiness (control, commitment and challenge), perceived social support, chronological age, sex (with men as the reference group), and competitive category (youth athletes under 23 years of age vs. seniors aged 23 years and above). Furthermore, an interaction term between competitive category and social support was included to examine whether the effect of social support on burnout varied between the category groups. The results are shown in Table 3.

**Table 3:** Multiple enter linear regression models.

Model	Reduced sense of accomplishment			Emotional exhaustion			Devaluation			Burnout		
	*B*	*β*	*t*	*B*	*β*	*t*	*B*	*β*	*t*	*B*	*β*	*t*
Intercept	24.38		18.27***	28.89		17.84***	22.17		12.90***	75.43		21.59***
Age	−0.05	−0.17	−2.37*	−0.03	−0.08	−1.09	−0.01	−0.02	−0.30	−0.08	−0.11	−1.56
Control	−0.17	−0.20	−3.76***	−0.24	−0.22	−4.24***	−0.08	−0.07	−1.27	−0.49	−0.21	−4.03***
Commitment	−0.11	−0.12	−2.23*	−0.25	−0.21	−4.10***	−0.24	−0.22	−3.73***	−0.59	−0.24	−4.59***
Challenge	−0.07	−0.10	−1.92	−0.15	−0.15	−3.14**	−0.00	−0.00	−0.06	−0.22	−0.11	−2.22*
Social support	−0.10	−0.19	−2.35*	−0.18	−0.26	−3.45***	−0.08	−0.13	−1.46	−0.37	−0.25	−3.21**
Sex	−0.21	−0.09	−0.94	0.50	0.16	1.86	−0.34	−0.12	−1.19	−0.05	−0.01	−0.08
Category	−2.10	−0.20	−1.82	−3.63	−0.19	−2.59*	−2.79	−0.15	−1.87	−8.52	−0.22	−2.82**
Social suppot × Category	0.08	0.15	1.52	0.15	0.21	2.34*	0.12	0.18	1.71	0.35	0.23	2.51*

Sex, male as reference; Category, young as reference; B, Unstandardized coefficient; β, Standardized coefficient.

* *p* < .05.

** *p* < .001.

The resulting models predicted the following percentages of the variance of the respective variables. Reduced satisfaction sense, 22% [*F*(8, 379) = 13.47, *p* < 0.001]; emotional exhaustion, 30% [*F*(8, 379) = 20.67, *p* < 0.001]; devaluation, 10% [*F*(8, 379) = 5.37, *p* < 0.001]; and burnout, 30% [*F*(8, 379) = 20.15, *p* < 0.001].

**Figure 1:**
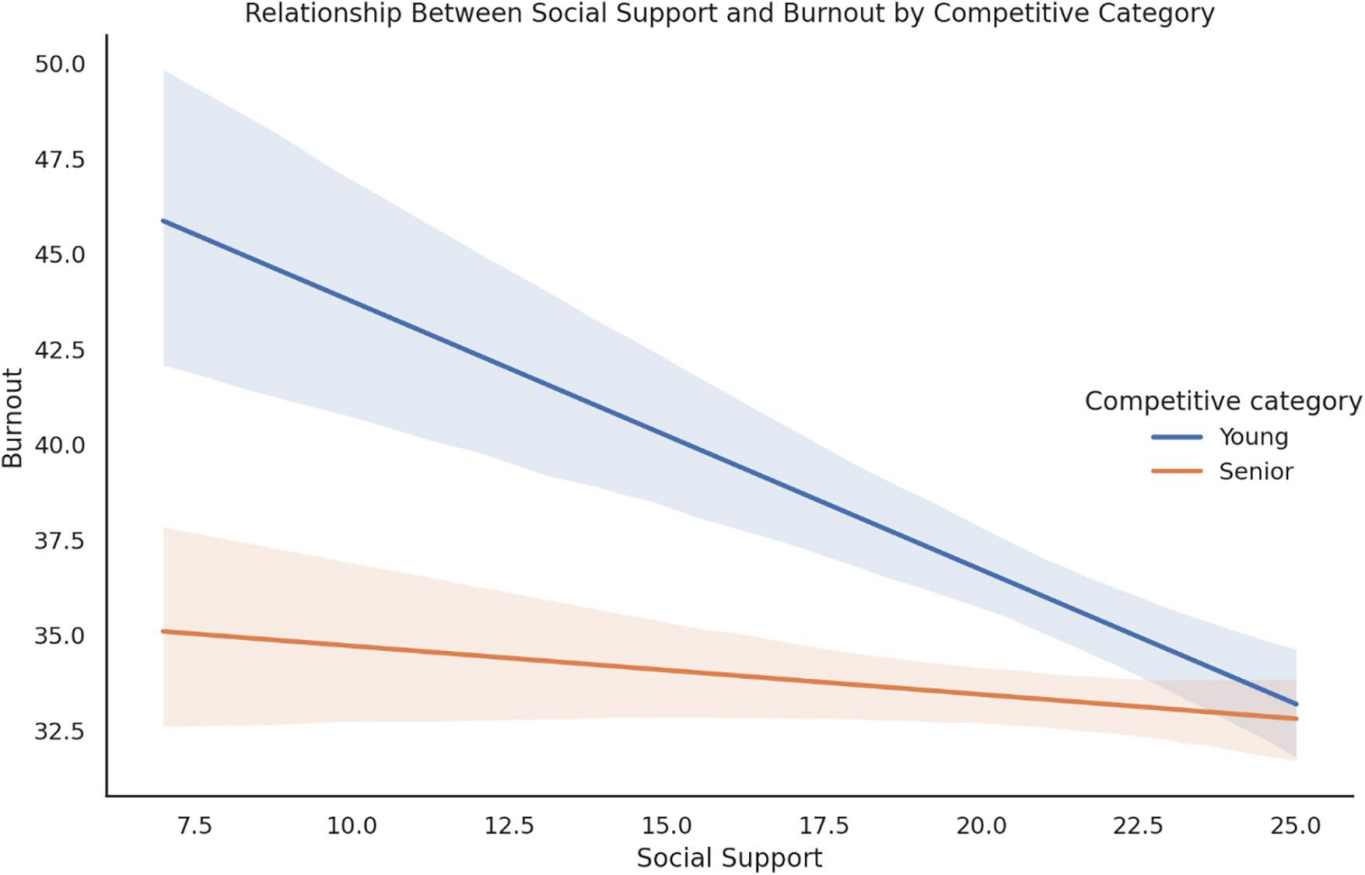
Interaction between social support and competitive category in predicting total burnout.

Figure 1 illustrates the interaction effect between social support and the competitive category in predicting total burnout. The graph shows that the negative association between social support and burnout was stronger among youth athletes than among senior athletes.

## 4 Discussion

The findings of this study indicate that both psychological hardiness and perceived social support play relevant roles in explaining burnout levels among high-performance athletes. This directly addresses the general objective of examining whether these variables may protect against or moderate burnout indicators. While both constructs have independently shown beneficial effects on athlete well-being in prior studies (47–51), their combined influence on burnout had not yet been examined. Therefore, this research contributes novel evidence by exploring the simultaneous effects of both variables across key dimensions of athlete burnout, helping clarify how individual psychological resources and interpersonal support interact in highly demanding sport contexts.

Aligned with the objective of this study that included examining differences in burnout components across demographic characteristics, the results showed that sex was not a significant factor in any burnout component. According to primary studies, burnout is more common among women (6). However, the nature of the gender–burnout relationship remains inconclusive, as few studies have examined it directly and empirical results have been inconsistent [for example, (52–54)]. This finding is consistent with Alves dos Santos et al. (55) and aligns with recent studies that have questioned the consistency of sex-related differences in athlete burnout, since no strong linear relationships have been found between sex and burnout indicators (56). Overall, this suggests that burnout may be more influenced by psychological and contextual variables (57, 58) than by sex.

Regarding the objective of exploring the influence of age and competitive category on burnout, the results of the Kruskall–Wallis test in the present study show that older athletes reported lower levels of emotional exhaustion, reduced sense of accomplishment, devaluation, and general burnout than younger athletes. These findings contrast with those of previous studies that found no significant association between age and burnout (56). Additionally, data suggest that competitive experience, operationalized through the competition level—may serve as an additional protective factor, potentially reflecting greater psychological maturity, coping resources, and resilience developed over time. Although Holden et al. (53) found no significant correlations between years of sports competition and the three burnout components, it should be noted that the samples in the different sports were very small. Furthermore, the level of competition is an indicator of devaluation: the higher the level of competition, the lower the likelihood of experiencing feelings of devaluation and a diminished sense of the importance in athletes' lives.

Corresponding to the objective of examining the associations between psychological hardiness and burnout, the results showed that hardiness, as a personal trait, emerged as the most relevant psychological predictor in all linear regression models. Its three dimensions, control, commitment, and challenge, were negatively associated with burnout levels and contributed significantly to the prediction of burnout. Among these, commitment has shown the most consistent and substantial influence, highlighting its role in maintaining motivation and engagement in sports (59). In university students and wrestling coaches (60), hardiness was negatively correlated with athletic burnout, suggesting that psychologically hardy athletes are less prone to burnout symptoms (61). Taken together, these findings demonstrate the protective role of hardiness in mitigating burnout in high-performance athletes. However, in the case of devaluation, only competition level and commitment were significant predictors, indicating that commitment to the sport plays an important role in the perception of value and meaning within it, as Raedeke (62) found in his primary research. The devaluation of sports, understood as a situation in which sports practice is no longer as rewarding as it once was, is a relevant factor in the appearance of burnout (63). According to the results of this study, commitment serves as a protective factor, which means that a higher commitment is associated with a lower probability of burnout, especially when it is an enthusiastic commitment (64).

In agreement with the aim of examining the relationship between perceived social support and burnout, social support also contributed to predicting emotional exhaustion and total burnout. Other studies have found similar results for emotional and physical exhaustion (65). In addition, a significant interaction between social support and the level was identified, indicating a moderating effect. Specifically, the protective impact of social support on burnout was stronger in youth athletes than in senior athletes (66). This suggests that athletes in the early competitive stages may rely more heavily on interpersonal resources to mitigate burnout symptoms.

### 4.1 Conclusions

In direct alignment with the objectives of this study, although previous studies have examined hardiness, social support, and level of competition as separate predictors of burnout, this study is among the first to evaluate their combined and simultaneous influences in burnout dimensions. This integrated approach allows for a more comprehensive understanding of the relative weight of each factor and how they interact in athletic contexts. In the model that predicts burnout, all variables, except sex, made significant contributions. The hardiness dimensions explained the largest proportion of variance across the models. The inclusion of social support and competition level added predictive value, but to a lesser extent than the other variables. This finding reinforces the central role of personal psychological resources in understanding athlete burnout. These findings may inform the development of multifactorial interventions tailored to different stages of athletic development in the future.

This study explored the different pathways through which psychological factors influence high-performance outcomes in athletes, highlighting the interaction between cognitive, emotional, and motivational processes in competitive contexts. We can assume that optimal performance is not merely a function of physiological preparedness or technical ability, but results from the complex integration of psychological, personal, and contextual variables. This multidimensional perspective aligns with contemporary sports psychology frameworks that view performance excellence as an emerging property of dynamic person–environment interactions rather than the product of isolated traits. It is also important to note that performance excellence can be conceptualized and facilitated with a model to develop service delivery programs (67) such as the CAC model or included in a Psychological Skill Training program.

These results therefore provide theoretical support for holistic frameworks in sports psychology, where cognitive appraisal, emotional balance, and motivational alignment are combined to sustain optimal functioning. This integrative approach challenges reductionist paradigms and supports the ongoing evolution of sports psychology toward multidimensional and context-sensitive models.

### 4.2 Limitation study and future directions

However, this study has several limitations that must be acknowledged. The cross-sectional nature of the data constrains causal inference, and future research employing longitudinal or experimental designs could clarify the temporal dynamics of the psychological adaptation. Additionally, expanding the sample to include diverse cultural and sporting contexts would improve the ecological validity of the findings and allow for more nuanced comparisons across disciplines in the future. Further investigation using mixed methods approaches, combining psychometric assessment, qualitative interviews, and physiological markers, would deepen our understanding of how psychological skills are internalized and maintained across competitive cycles.

### 4.3 Practical implications

Therefore, interventions aimed at preventing burnout should be designed with a focus on individual needs (68, 69) rather than sex-based assumptions. Perceived social support plays a key role in sustaining athletes' engagement in sport and reducing vulnerability to burnout, particularly during stressful periods such as poor performance in major competitions (51). Coaches are encouraged to regularly assess athletes for signs of burnout using standardized tools, such as the Athlete Burnout Questionnaire (ABQ), for consistent monitoring. In terms of intervention in extremely severe cases of burnout, short-term psychotherapy, family, and federation support in combination have been of great importance for an Olympic swimmer (1), and shows the importance that in serious cases, a psychologist with clinical experience is desirable, as Weinberg and Gould (70) have shown when distinguishing between types of sports psychologist: performance and clinical.

Additionally, open communication should be promoted, especially with respect to the dimensions of commitment and control, as they have been shown to reduce emotional exhaustion. Furthermore, promoting diversification by encouraging older athletes to mentor younger ones can help foster the understanding that training and competing are challenging processes, but with commitment, control and a sense of challenge, success in sports can be achieved. Particular attention should be paid to young athletes, who may be more receptive to psychological interventions and benefit more from increased social support networks, as they appear more vulnerable to burnout. Finally, scientific evidence underscores the importance of coaches being open to facilitating both direct and indirect psychological support when necessary, emphasizing the central role of mental health in maintaining athletic performance in sports.

## Data Availability

The raw data supporting the conclusions of this article will be made available by the authors, without undue reservation.
